# The effect of succinic acid on the metabolic profile in high‐fat diet‐induced obesity and insulin resistance

**DOI:** 10.14814/phy2.14630

**Published:** 2020-11-13

**Authors:** Stephen J. Ives, Kendall S. Zaleski, Cheyanne Slocum, Daniela Escudero, Caty Sheridan, Saada Legesse, Kavey Vidal, Sarita Lagalwar, Thomas H. Reynolds

**Affiliations:** ^1^ Health and Human Physiological Sciences Skidmore College Saratoga Springs NY USA

**Keywords:** metabolism, mitochondrial function, obesity, type 2 diabetes

## Abstract

Obesity, insulin resistance, and poor metabolic profile are hallmarks of a high‐fat diet (HFD), highlighting the need to understand underlying mechanisms. Therefore, we sought to determine the effect of succinic acid (SA) on metabolism in high‐fat diet (HFD)‐induced obesity. Animals were randomly assigned to either low‐fat diet (LFD) or a high‐fat diet (HFD). Mice consumed their respective diets for 4.5 months and then assigned to the following groups: (LFD)+vehicle, LFD + SA (0.75 mg/ml), HFD + vehicle, or HFD + SA. Body weight (BW), food, and water intake, were tracked weekly. After 6 weeks, insulin, glucose, and pyruvate tolerance tests were completed, and spontaneous physical activity was assessed. Epididymal white adipose tissue (EWAT) mass and in vitro measurements of oxidative skeletal muscle (soleus) respiration were obtained. Expectedly, the HFD increased BW and EWAT mass, and reduced glucose and insulin tolerance. SA significantly reduced EWAT mass, more so in HFD (*p* < .05), but had no effect on any in vivo measurements (BW, insulin, glucose, or pyruvate tolerance, nor physical activity, all *p* > .05). A significant (*p* < .05) interaction was observed between mitochondrial respiration and treatment, where SA increased respiration, likely owed to greater mitochondrial content, as assessed by complex IV activity in both LFD and HFD. In HFD‐induced obesity, coupled with insulin desensitization, we found no favorable effect of succinic acid on glucose regulation, though adiposity was attenuated. In oxidative skeletal muscle, there was a tendency for increased respiratory capacity, likely owed to greater mitochondrial content, suggestive of a succinic acid‐induced mitochondrial biogenesis.

## Graphical abstract

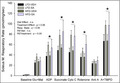

**New & Noteworthy**

Obesity, insulin resistance, and poor metabolic profile are hallmarks of a high‐fat diet (HFD), highlighting the need to understand underlying mechanisms and potential treatments. In the HFD‐induced model of obesity, with marked insulin resistance, we treated mice with succinic acid, a metabolite, and complex II agonist. Succinic acid had no positive effect on glucose regulation, but seemed to reduce adiposity, and increase mitochondrial respiratory capacity in oxidative skeletal muscle, likely due to a succinic acid‐induced mitochondrial biogenesis.

## 1 INTRODUCTION

Over the last several decades, the occurrence of obesity in the United States, and worldwide, has been progressively increasing. Between 1980 and 2013, the worldwide rate of overweight and obese adults increased by nearly 30% during this period, for a total of 2.1 billion individuals experiencing obesity and being overweight (Apovian, 2016) In the United States alone, it is estimated that more than 68% of US adults are classified as overweight (BMI of 25.0–29.9 kg/m^2^) and 35% are considered obese (BMI <30 kg/m^2^; Flegal et al., 2010). The major contributors to the rising overweight occurrence are the consumption of high‐calorie and/or fatty diets and a sedentary lifestyle both of which can lead to the onset of obesity, insulin‐resistant states such as pre‐diabetes, and can ultimately lead to the development of type 2 diabetes and other co‐morbidities (Shortreed et al., 2009; Trajcevski et al., 2013).

Skeletal muscle, which is the greatest tissue mass in the body, plays an important role in insulin‐stimulated glucose disposal (Deshmukh, 2016; Wu & Ballantyne, 2017) and lipid oxidation (Kelley et al., 1999). Under normal physiological conditions, skeletal muscle is responsible for most insulin‐stimulated glucose disposal in the body; therefore, skeletal muscle dysfunction due to obesity can robustly impact glucose homeostasis and insulin sensitivity (Wu & Ballantyne, 2017); thus, possibly leading to a diabetic state (Deshmukh, 2016). The high glucose disposal rate of skeletal muscle, and high capacity to adapt to metabolic demands (Hood, 2001), are associated with relatively high mitochondrial content (Hood, 2001; Leskinen et al., 2010). In obesity, decreased activity of oxidative enzymes associated with ATP synthase and mitochondrial content has been reported (Kelley et al., 1999), suggesting that a HFD induces mitochondrial dysfunction, perhaps specific to complex II (Ngo et al., 2019). Thus, skeletal muscle is a prime target to understand the impact of underlying factors, such as diet, in mitochondrial dysfunction, and impaired glucose tolerance, as well as exploring potential novel interventions.

Various diseased states, such as type 2 diabetes (Gupte et al., 2013; Larsen et al., 2009), chronic obstructive pulmonary disease (Gifford et al., 2015), heart disease (Park et al., 2014), and neurodegenerative diseases (Ferro, Carbone, Marzouk, et al., 2017; Ferro, Carbone, Zhang, et al., 2017), have been demonstrated to negatively impact mitochondrial function. Additionally, a high‐fat diet (McCreath et al., 2015; Özyazgan et al., 1998) and obesity (Gupte et al., 2013; Shortreed et al., 2009) have also been shown to cause intrinsic mitochondrial dysfunction. Due to the impact of chronic diseases, a high‐fat diet, or obesity on mitochondrial function, more research needs to be performed to explore the functional properties of mitochondria and its oxidative phosphorylation capacity (Kuznetsov et al., 2008; Larsen et al., 2012). Dysfunction of the mitochondria can lead to excessive free‐radical production (Gifford et al., 2015; Pari & Saravanan, 2007; Park et al., 2014; Ungvari et al., 2010), decreased oxidative phosphorylation efficiency (Ferro, Carbone, Zhang, et al., 2017; Park et al., 2014; Shortreed et al., 2009), and reduced mitochondrial ATP production due to interruptions in the electron transport chain (Ferro, Carbone, Zhang, et al., 2017; Gifford et al., 2015; Gupte et al., 2013; Park et al., 2014). Conversely, there have been varying reports of decreased (Chanseaume et al., 2006), increased (Pari & Saravanan, 2007; Shortreed et al., 2009), and unaffected (Koves et al., 2008) oxidative capacity in high‐fat diet‐induced obesity, highlighting the need for further work.

Given the role mitochondrial dysfunction may play in obesity and insulin resistance, there has been increased focus on interventions (i.e. antioxidants, Statins, etc.) to mitigate this process (Gioscia‐Ryan et al., 2014, 2018; Ungvari et al., 2010). However, there are concerns as to whether antioxidants might actually reduce mitochondrial biogenesis (Gomez‐Cabrera et al., 2008; Strobel et al., 2011) and thus mitochondrial function, raising the importance of finding potential alternatives to improving mitochondrial function. To this end, succinic acid, a Krebs cycle intermediate that activates complex II of the ETC, via succinate dehydrogenase‐induced generation of FADH_2_, is an FDA‐approved compound used in food/drug processing that has been suggested as a potential therapeutic (British National Formulary for Children). This may be particularly relevant in obesity which has been suggested to have a deficiency in complex II (Ngo et al., 2019).

Treatment with succinic acid analogs, such as succinic acid monoethyl ester, have been shown to have insulinotropic (MacDonald & Fahien, 1988; Özyazgan et al., 1998) and antioxidant properties (Pari & Saravanan, 2007; Saravanan & Pari, 2006), resulting in lowering of blood glucose, glycosylated hemoglobin, and improved redox balance in a murine model of type 1 diabetes (Pari & Saravanan, 2007; Saravanan & Pari, 2006). In addition, exogenous succinic acid treatment has indicated improvements to motor behavior (Ferro, Carbone, Zhang, et al., 2017), amelioration of cognitive deficits (Ferro, Carbone, Zhang, et al., 2017; Storozheva et al., 2008), and mitigated mitochondrial oxidative phosphorylation dysfunction (Ferro, Carbone, Zhang, et al., 2017; Storozheva et al., 2008) in animal models of neurodegenerative diseases. Sustained elevations in succinate, and subsequent activation of the G protein‐coupled receptor 91 (GPR91) or succinate receptor (SUCNR1), may also be indicative of mitochondrial stress due to hypoxia, diabetes, or cancer (Peti‐Peterdi, 2010). In obesity, succinate, via SUCNR1, may enhance or impair macrophage inflammation (Keiran et al., 2019; van Diepen et al., 2017), leaving it unclear whether exogenous succinate might be beneficial in HFD‐induced obesity. However, no work, to date, has explored the potential impact of SA on a HFD‐induced model of obesity and insulin resistance, so this could be important as the treatment may ameliorate complex II‐associated mitochondrial dysfunction, improving oxidative metabolism, and thus possibly diminishing the metabolic effects of the disease.

Accordingly, the purpose of the study was to examine the impact of succinic acid on body weight, adiposity, insulin action, and mitochondrial function in high‐fat diet‐induced obesity, and associated insulin resistance, in C57/Bl6 male mice. It was hypothesized that exogenous treatment with succinic acid, a complex II electron donor, would have therapeutic effects (i.e., improved body weight, reduced adiposity, improved insulin action/glucose tolerance, etc.) in high‐fat diet‐induced obese mice through improved mitochondrial oxidative phosphorylation capacity.

## 2 METHODS

### 2.1 General procedures

**FIGURE 1:**
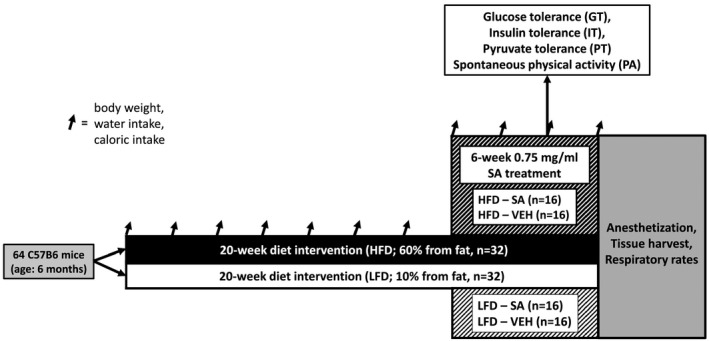
Experimental overview. Experimental groups included high‐fat diet succinic acid‐treated (HFD‐SA), high‐fat diet vehicle (HFD‐VEH), low‐fat diet succinic acid‐treated (LFD‐SA), and low‐fat diet vehicle (LFD‐VEH)

64 male C57B/6J mice (1 month old) were obtained from Jackson Laboratory. Animals were housed at the Skidmore College animal facility on a 12:12 light:dark cycle and had access to food and water ad libitum. After initial acclimatization (1 week), the mice were randomized into either a high‐fat diet (HFD; 60% kcal from fat) or a low‐fat diet (LFD; 10% kcal from fat) group and consumed their respective diet for 4.5 months (Test Diets; Bloom et al., 2020; Reynolds et al., 2019; Shortreed et al., 2009; Trajcevski et al., 2013). Using a randomized controlled design, during the final 8 weeks of the diet intervention, the groups were subsequently randomized to receive either vehicle (VEH, drinking water) or succinic acid (SA; 0.75 mg/ml in drinking water; Ferro, Carbone, Marzouk, et al., 2017; Ferro, Carbone, Zhang, et al., 2017; Figure 1). Body weight, food, and water intake were assessed throughout the treatment period. Body mass was measured using a digital lab scale each week. Caloric and succinic acid intake were measured by providing mice a known amount of food and water for a given time period (1–4 days) and the residual chow and water were weighed and subtracted from the initial amount. The quantity of food intake was multiplied by the kcal/g for each diet and value expressed as kcal/day. Succinic acid supplementation was conducted as described in Ferro et al. (Ferro, Carbone, Marzouk, et al., 2017; Ferro, Carbone, Zhang, et al., 2017). An overview of the experimental design is displayed in Figure 1. Guidelines set forth by the National Research Council's Guide for Care and Use of Laboratory Animals (Institute of Laboratory Animal Resources, Commission on Life Sciences, 2011) were followed, and experimental protocols were approved by the Skidmore College Institutional Animal Care and Use Committee (Protocol #123).

### 2.2 In vivo testing

During the last 2 weeks of SA treatment, measures of basal fasting glucose and lipid profile, glucose tolerance (GT), insulin tolerance (IT), and spontaneous physical activity (PA), assessed via voluntary wheel running for 24 hr, were obtained. For basal fasting glucose and lipids approximately 40 µl of fasting venous blood was collected for cholesterol analysis. The concentrations of glucose, total cholesterol, triglycerides, high‐density lipoprotein‐cholesterol, low‐density lipoprotein‐cholesterol, and non‐high‐density lipoprotein‐cholesterol were assessed using the standard enzymatic technique (Cholestech LDX Analyzer, Alere; DeFronzo & Tripathy, 2009).

To test glucose tolerance, the mice completed an overnight fast prior to receiving an intraperitoneal injection of glucose (2.0 g/kg body weight; Reynolds et al., 2019). Subsequently, venous blood from the tails was obtained (~5 µl) at 15, 30, 45, 60, 90, and 120 min post‐injection and blood glucose was assessed using a hand‐held glucometer (Accu‐Check, Roche Diabetes Care, Inc; Reynolds et al., 2019). In addition to the glucose tolerance test, insulin tolerance testing was also performed to assess insulin action. For the insulin tolerance test, the glucose tolerance testing procedure was replicated with the alteration that the mice received an intraperitoneal injection of insulin (0.75 U/kg body weight) following a 6‐hour fast (Reynolds et al., 2019). Finally, to assess potential differences in gluconeogenesis with diet and SA treatment in HFD, a pyruvate tolerance test was completed via intraperitoneal injection of pyruvate following a 6‐hour fast. Mice were recovered between each tolerance test.

### 2.3 Tissue harvest

Upon the completion of the in vivo testing, mice were anesthetized with a 1:1:1 mixture of PromAce, ketamine hydrochloride, and xylazine by intraperitoneal injection (1.5 ml/kg; Bloom et al., 2020  ). The soleus muscle, an oxidative skeletal muscle, was dissected and removed for subsequent determination of in vitro mitochondrial respiratory capacity. The EWAT were excised intact and rapidly weighed using a digital scale, as previously described (Reynolds et al., 2019), as a marker of adiposity. In our laboratory, EWAT mass displays a high degree of correlation with more sophisticated measures of fat mass, such as magnetic resonance spectroscopy (*r* = .97, *p* < .0001, *n* = 21, unpublished observations). After the removal of tissues, unconscious mice were euthanized by cervical dislocation. Permeabilized whole soleus tissue was used to assess oxidative capacity instead of isolated mitochondria because previous studies have found whole tissue preparations prevent unhealthy, decreased density and activity, or damaged mitochondria in the respiratory analysis (Kuznetsov et al., 2008; Shortreed et al., 2009). The dissected soleus was placed in a chilled buffer A (in mM: 2.8 CaK_2_EGTA, 7.2 K_2_EGTA, 6.6 MgCl_2_, 0.5 dithiothreitol [DTT], 50 K‐MES, 20 imidazole, 20 taurine, 5.3 Na_2_ATP, 15 phosphocreatine, pH 7.3 at 4°C) and the sample remained in this solution until further dissection and permeabilization (<30 min) (Park et al., 2014). To ensure adequate diffusion of the permeabilizing agent and substrates, soleus muscle was finely teased by needle tip. Afterward, to chemically permeabilize fibers, samples underwent mild shaking for 30–40 min in buffer A with saponin (50 mg/ml), and the muscle was rinsed twice in buffer B (in mM: 2.8 CaK_2_EGTA, 7.2 K_2_EGTA, 1.4 MgCl_2_, 3.0 K_2_HPO_4_, 0.5 dithiothreitol, 20 imidazole, 100 K‐MES, 20 taurine, pH 7.3 at 4°C) for 10 min.

### 2.4 Mitochondrial respiration

Mitochondrial respiration experiments were modified from previous studies (Ferro, Carbone, Marzouk, et al., 2017; Ferro, Carbone, Zhang, et al., 2017; Kuznetsov et al., 2008; Park et al., 2014). Mitochondrial respiratory oxygen flux (*J*O_2_) was assessed using a substrate and inhibitor protocol with a high‐resolution Clark‐type Oxygraph respirometer (Oxytherm, Hansatech). The permeabilized muscle fibers were incubated in the respirometer with 2 ml of buffer B, while being continuously stirred at 37°C. First, baseline muscle respiration was recorded, in the absence of respiratory substrates. To assess the function of each mitochondrial complex, oxygen consumption was assessed with the addition of a series of respiratory substrates and inhibitors in the following order and concentration: glutamate‐malate (2000:800 mM), ADP (500 mM), succinate (1000 mM), cytochrome c (4 mM), rotenone (0.2 mM), oligomycin (4 mg/ml), antimycin‐A (5 mM), N,N,N,N‐tetramethyl‐ p‐phenylenediamine (TMPD)‐ascorbate (200:800 mM) using glass micro syringes. This protocol, as described by Kuznetsov et al. (2008), allowed the determination of (a) complex I state 2 respiration, the non‐phosphorylating resting state that provides an index of proton leak, assessed in the presence of malate + glutamate, (b) Complex I, state 3 respiration, the ADP‐activated state of oxidative phosphorylation, assessed in the presence of glutamate + malate +ADP, (c) Complex I + II, state 3 respiration, assessed in the presence of glutamate + malate +succinate + ADP, (d) Complex IV, experimentally induced by the addition of TMPD + ascorbate. In each condition, respiration rate was recorded for 3 min and the average of the last min was used for data analysis. Mitochondrial membrane damage was evaluated by cytochrome c induction. The rate of oxygen consumption was measured in pmol of O_2_ per second and then expressed relative to muscle sample mass (pmol/sec/wet weight). All chemicals were obtained from Sigma‐Aldrich.

### 2.5 Data and statistical analysis

Assessments of statistical significance differences by single effect (LFD vs. HFD) were analyzed using independent samples *t* tests. Comparisons of multiple effects, namely diet (LFD vs. HFD), treatment (VEH vs. SA), time, and their potential interactions were analyzed using analysis of variance (ANOVA). In the case of GTT and ITT, focusing on the target hypothesis of SA treatment improving HFD‐induced insulin resistance, in parallel with resource restraints, we targeted testing the LFD‐VEH, HFD‐VEH, and HFD‐SA, and thus these data were analyzed by group (LFD, HFD‐VEH, and HFD‐SA) over time using a two‐way (group x time) ANOVA. Tests of normality were conducted, and if a significant violation was found adjustments were made to the degrees of freedom using the Greenhouse‐Geisser correction. Statistical analyses were conducted in commercially available software (SPSS v.26, IBM). Significance was established at *p* < .05. Data, where indicated, are presented as mean ± standard deviation (*SD*).

## 3 RESULTS

### 3.1 Diet, body weight, adiposity, and physical activity

**FIGURE 2:**
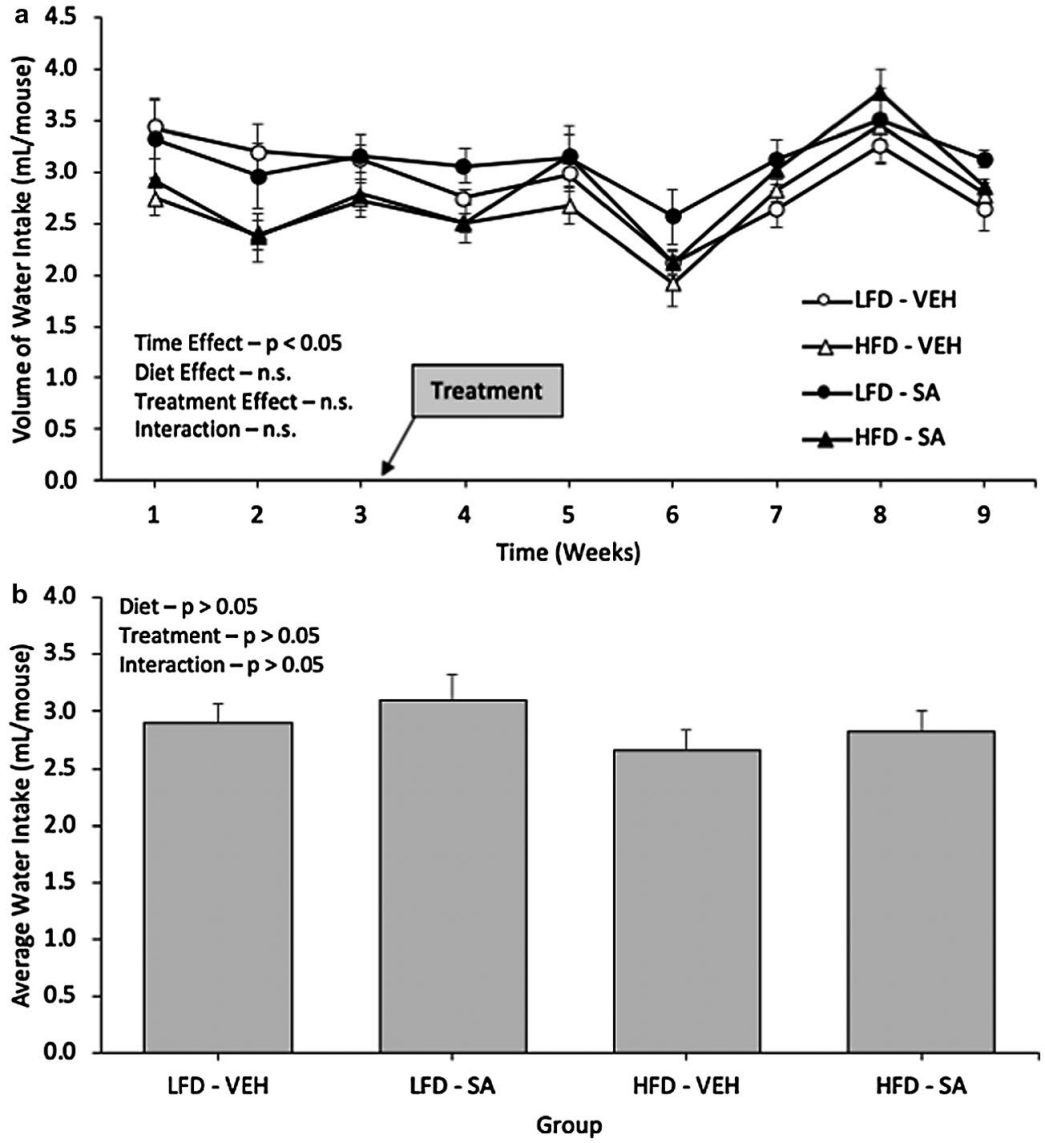
Volume of water intake (a) and average water intake (b) in low‐fat diet (LFD) or high‐fat diet (HFD) Mice treated with vehicle (VEH) or Succinic Acid (SA). Data are mean ± *SD*

To evaluate the average amount of succinic acid intake, the volume of water consumption over the treatment period was assessed (Figure 2). In terms of water intake, and thus succinic acid, while consumption tended to vary over time (*p* < .05), there were no significant interactions of diet or treatment over time (*p* > .05) or main effects of diet or treatment alone (Figure 2a). Thus, mice treated with SA consumed water in a manner similar to the vehicle. On average, intake did not differ significantly with diet (*p* > .05) or treatment (*p* > .05), nor was there a significant interaction (*p* > .05) in water intake (Figure 2b). Finally, to directly compare the SA‐treated groups, the average succinic acid intake of the LFD‐SA mice had an average of 4.1 ± 0.2 mg SA intake and the HFD‐SA mice had a mean SA intake of 3.8 ± 0.3 mg over the treatment period (*p* > .05).

**FIGURE 3:**
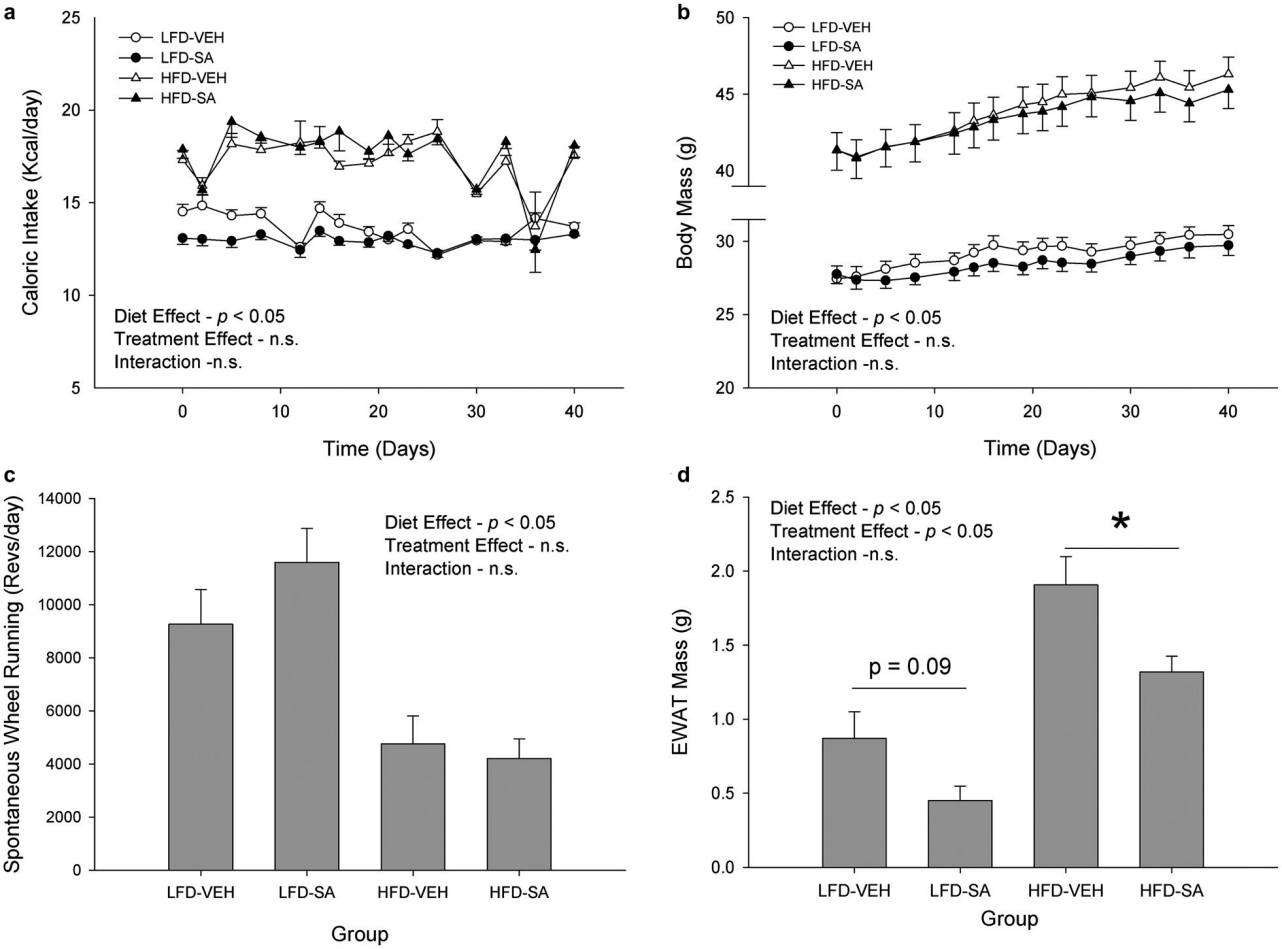
Caloric intake (a), body mass (b), spontaneous wheel running (c), and epididymal white adipose tissue mass (d) in low‐fat (LFD) or high‐fat diet (HFD) mice treated with vehicle (VEH) or succinic acid (SA). *p < .05 HFD‐VEH versus HFD‐SA. Data are mean ± *SD*

To determine if SA treatment altered the effects of a high‐fat diet on adiposity and energy homeostasis, we assessed caloric intake, body mass, physical activity, and epididymal white adipose tissue (EWAT) mass (Figure 3). For caloric intake, a significant diet effect was observed (*p* < .05), yet there were no significant interactions of diet and treatment over time nor the main effects of treatment alone (*p* > .05, Figure 3a). As expected, the high‐fat diet mice had higher rates of caloric consumption than the low‐fat diet mice. With regard to body mass, there was a significant main effect for diet (*p* < .05) where HFD mice had significantly larger body masses compared to the LFD mice (Figure 3b). There was no significant effect for the SA treatment or an interaction of diet and treatment over time (*p* > .05) found for body mass (Figure 3b). The mice's diet had a significant effect (*p* < .05) on physical activity, assessed via spontaneous wheel running, with the LFD mice completing more revolutions on the wheel per day compared to the HFD mice; however, there was no significant effect of the SA treatment or an interaction of diet or treatment (*p* > .05, Figure 3c). Finally, EWAT mass was assessed on a digital scale after excision. There was a significant effect of both diets (*p* < .05) and treatment alone (*p* < .05) on the EWAT masses of the mice; however, there was no significant interaction observed (*p* > .05, Figure 3d). The high‐fat diet mice had a significantly higher EWAT weight than the low‐fat diet mice. Meanwhile, the SA‐treated mice had significantly lower EWAT masses than the vehicle mice, which was more pronounced in HFD (*p* < .05) than LFD (*p* = .09).

### 3.2 Glucose, insulin, and pyruvate tolerance

**FIGURE 4:**
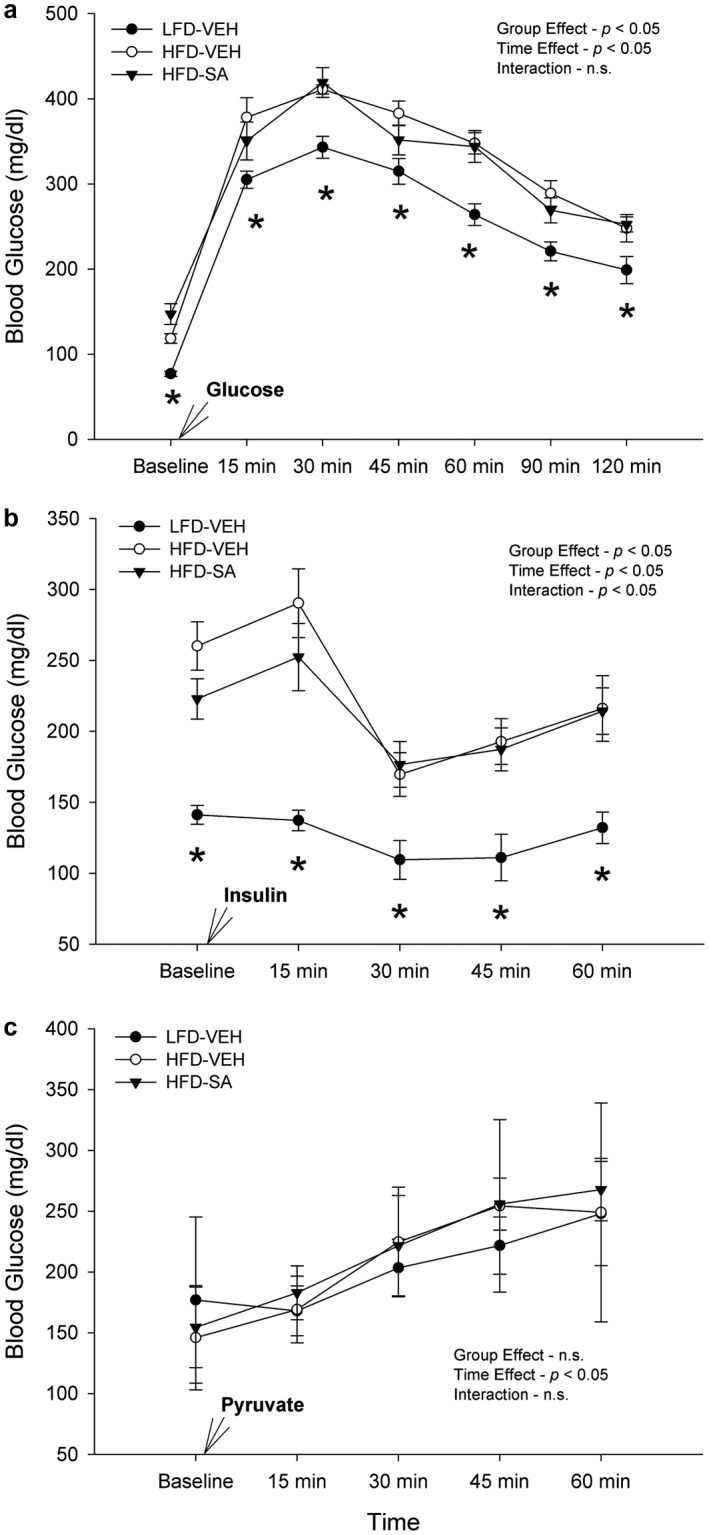
Glucose tolerance test (a), insulin tolerance test (b), and pyruvate tolerance test (c) in low‐fat diet (LFD) or high‐fat diet (HFD)‐fed mice treated with vehicle (VEH) or succinic acid (SA). *p < .05 HFD‐VEH versus HFD SA. Data are mean ± *SD*

To assess the role of diet and SA treatment on metabolic profile, measures of blood glucose and lipid content (Table 1), and glucose and insulin tolerance tests (Figure 4) were performed. With regards to the basal fasting blood glucose, there was a significant diet effect (*p* < .05), such that the high‐fat diet mice had significantly greater fasting blood glucose levels than the low‐fat diet mice (Table 1). In addition, a significant treatment effect was demonstrated (*p* < .05) between the vehicle and succinic acid‐treated mice with treatment increasing basal glucose levels, which was largely driven by the HFD (*p* < .05) as the effect in LFD was not significant (*p* > .05); however, no significant interaction between the diet or treatment was found (*p* > .05). Regarding glucose tolerance testing, a significant group effect and time effect were observed (*p* < .05) for the blood glucose data, yet there was no significant interaction between diet and treatment obtained (*p* > .05, Figure 4a). Insulin tolerance tests demonstrated a significant group effect (*p* < .05), time effect (*p* < .05), and interaction (*p* < .05) between group and time (Figure 4b), indicating that a HFD produced insulin resistance but SA had no effect. Finally, the pyruvate tolerance test revealed no significant group by time interaction (*p* > .05) or effect of group (*p* > .05) suggesting that a HFD and SA do not impact gluconeogenesis.

**TABLE 1:** Effects of diet and succinic acid treatment on fasting blood glucose and lipid profile

	LFD‐V	LFD‐SA	HFD‐V	HFD‐SA
Glucose (mg/dl)	78 (±3)	87 (±2)	119 (±5)^b^	147 (±11)^c^
TC (mg/dl)	113 (±5)	111 (±8)	117 (±4)	127 (±8)
HDL (mg/dl)	44 (±5)	42 (±4)	51 (±8)	54 (±2)
TRG (mg/dl)	68 (±8)	103 (±14)^a^	97 (±16)	93 (±26)
LDL (mg/dl)	59 (±5)	68 (±4)	36 (±3)^b^	50 (±5)^c^
non‐HDL (mg/dl)	78 (±4)	86 (±4)	57 (±3)^b^	73 (±9)
TC/HDL (mg/dl)	2.7 (±0.3)	2.7 (±0.4)	3.0 (±1.0)	2.4 (±0.2)

Note

Data are mean ± *SD*.

Abbreviations: HDL, high‐density lipoprotein; LDL, low‐density lipoprotein; non‐HDL, non‐high‐density lipoprotein; TC, total cholesterol; TC/HDL, total cholesterol to high‐density lipoprotein ratio; TRG, triglyceride.

^a^ *p* < .05 LFD‐VEH versus LFD‐SA.

^b^ *p* < .05 LFD‐VEH versus HFD‐VEH.

^c^ *p* < .05 HFD‐VEH versus HFD‐SA.

### 3.3 Blood lipids

Blood lipid content was analyzed through examining the total cholesterol (TC), low‐density lipoproteins (LDL), high‐density lipoproteins (HDL), non‐high‐density lipoproteins (non‐HDL), triglyceride (TRG) content, and TC/HDL ratio (Table 1). No significant diet, treatment, or interaction effects were observed in TC, HDL, and the TC/HDL ratio (Table 1). In terms of LDL, and thus non‐HDL, there was a significant effect for diet, with blood LDL/non‐HDL levels being on average lower in the LFD versus HFD fed mice (*p* < .05, Table 1). Succinic acid treatment led to on average higher LDL/non‐HDL levels than was observed with the vehicle control (*p* < .05, Table 1).

### 3.4 Mitochondrial respiration

**FIGURE 5:**
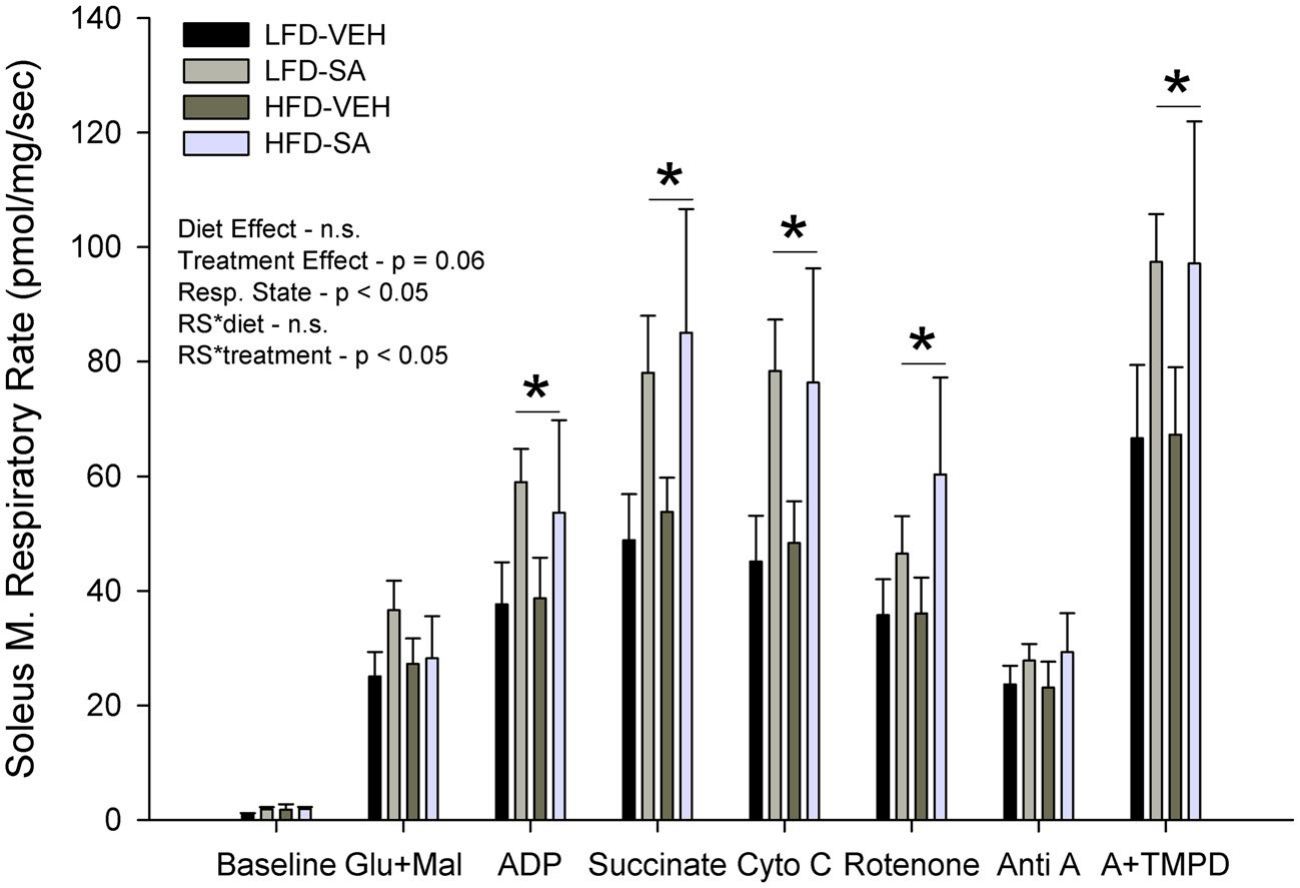
Mitochondrial respiratory capacity in low‐fat (LFD) or high‐fat diet (HFD) mice treated with vehicle (VEH) or succinic acid (SA). Glu + Mal = Complex I, (CI) State 2 (S2) respiration, ADP = CI S3, Succinate CI + II S3, Cyto C = check mitochondrial membrane integrity, Rotenone = CII S3, Anti A = C III inhibitor, A + TMPD = maximal C IV activity. Data are mean ± *SD*. *p < .05 SA treated versus VEH

Soleus muscle mitochondrial respiratory rate data were collected to determine the effect of a HFD and SA on mitochondrial function ex vivo. There was no observed significant effect of diet alone (*p* > .05), the interaction between diet and treatment (*p* > .05), or respiratory state and diet interaction (*p* > .05, Figure 5b). However, the interaction between respiratory state and treatment was significant (*p* < .05), such that as the respiratory rate increased, the succinic acid‐treated mice had a significantly high respiratory rate than the vehicle mice (Figure 5b). Furthermore, the main effect of treatment alone was trending to be significant (*p* = .06), with a tendency for greater respiratory rates with SA treatment. Subsequent analysis of each respiratory state revealed a significant impact of SA treatment on complex I driven, complex II driven, cytochrome c condition, and complex IV driven respiration (Figure 5). Baseline, leak (Glu + Mal), and complex III inhibition (AA) were not affected by SA treatment. Importantly, respiration rates were not significantly increased with the addition of cytochrome c, suggestive of an intact mitochondrial membrane and quality (*p* > .05). The A + TMPD response was significantly greater in muscles from SA‐treated animals (*p* < .05). As the A + TMPD response, indicative of mitochondrial content, differed with treatment, normalizing the respiratory rates to the A + TMPD on an individual basis abolished the interaction between respiratory state and treatment (*p* > .05), and thus there was no significant effect on treatment (*p* > .05) or diet (*p* > .05, data not shown).

## 4 DISCUSSION

The present study provides insight into the potential effects of succinic acid treatment on metabolic profile in high‐fat‐fed mice. The novel findings from the current study include: Succinic acid reduced adiposity, assessed via EWAT mass, particularly so in the mice fed a HFD. Succinic acid, unlike the previous work with the ester form, increased basal fasting blood glucose, but had no clear effect on glucose tolerance or insulin sensitivity. Succinic acid had no significant effect on spontaneous physical activity, assessed with wheel running. Last, succinic acid tended to increase mitochondrial respiratory capacity in oxidative muscle, likely owed to greater mitochondrial biogenesis, as indicated by the maximal complex IV activity. Collectively, these results suggest that succinic acid does not significantly impact body weight, metabolic profile, or physical activity, but is likely beneficial in terms of reducing adiposity, perhaps via mitochondrial biogenesis and elevated basal metabolism. Further work is needed to discern the possible metabolic consequences of chronic succinic acid treatment.

### 4.1 The impact of a HFD and SA treatment on body weight and adiposity

Obesity induced by a HFD is associated with amplified inflammation of adipose tissue depots and increases in adipose tissue mass (Keiran et al., 2019; Trajcevski et al., 2013; van Diepen et al., 2017). Expansion of adipose tissue following HFD consumption may, initially, have a positive effect as it allows for the sequestration of free fatty acids, thus reducing hyperlipidemia and subsequent adiposity in other tissues (Trajcevski et al., 2013). However, significantly increased epididymal fat pads lead to inflammation in the cells, furthering mitochondrial dysfunction, and glucose intolerance (van Diepen et al., 2017). In addition, it has been suggested that the succinate signaling pathway is involved in the movement of macrophages into adipose tissue, thereby impacting adipose tissue inflammation (Keiran et al., 2019; Trajcevski et al., 2013; van Diepen et al., 2017); however, the effect of succinate on the inflammatory processes of adipose tissue is dependent on physiological conditions (Mills et al., 2018). Similar to previous studies, our data revealed that both a HFD (Burcelin et al., 2002; Trajcevski et al., 2013) and succinic acid treatment (Keiran et al., 2019) had significant effects on EWAT weight, and thus is a good surrogate for visceral adiposity (Figure 3). Our data demonstrate that SA treatment using the present paradigm was effective at reducing EWAT mass in the male C57BL/6J mice, which suggests that diet‐induced obesity may respond to this treatment. While the SA treatment seemingly decreased adiposity, it was not clearly effective at reversing the overall body weight of the HFD mice, though it is interesting to note that body weights were on average ~5% lower with SA but this did not achieve statistical significance. These findings suggest that succinic acid might not a viable remedy for diet‐induced obesity, though further work that assesses body composition and regional fat distribution is warranted. Future work ought to explore the metabolic effects of chronic SA treatment upon adipose tissue itself, to elucidate the potential SA‐induced metabolic responses, adaptations, and underlying mechanisms in WAT, but also in other fat depots including brown adipose tissue (BAT).

### 4.2 The impact of SA treatment on physical activity and caloric intake in diet‐induced obesity

Previous studies have found that exercise increases the production of reactive oxygen species in skeletal muscle, which contributes to mitochondrial dysfunction (Strobel et al., 2011) and is associated with oxidative stress (Ungvari et al., 2010), obesity, and type 2 diabetes (Reynolds et al., 2019). However, endurance exercise training also promotes adaptations to cellular processes in skeletal muscle, such as improvements to insulin sensitivity and an increase in mitochondrial biogenesis which results in mitochondrial content and density (Larsen et al., 2012; Strobel et al., 2011). These adaptations may be correlated with the elevated levels of circulating succinate that is associated with endurance‐training exercise (Hochachka & Dressendorfer, 1976). The results from our study demonstrate that the HFD mice performed significantly less activity than the LFD mice, but there was no effect of succinic acid on the physical activity levels, suggesting that the observed reduction in adiposity was not the result of increased spontaneous physical activity (Figure 3). In contrast to our results, previous studies have suggested that pre‐diabetic mice, or HFD‐induced obese mice, may have an impaired ability to respond to exercise and immediately recover force following fatiguing muscle contractions, likely due to the weakened metabolic capacity of HFD muscles (Shortreed et al., 2009). Further, caloric intake was relatively stable and did not differ with treatment (Figure 3), suggesting that reductions in EWAT mass with SA were unlikely due to altered appetite and subsequent dietary intake. The lack of a treatment effect in our data suggests that increases in circulating succinic acid may not be effective in treating HFD‐induced obesity, or at least not through altered physical activity or caloric intake.

### 4.3 The impact of SA treatment on metabolic profile in diet‐induced obesity

As previously stated, HFD‐induced obesity causes an expansion of adipose tissue mass. This increase in adiposity is associated with glucose intolerance and impaired lipid oxidation (Shortreed et al., 2009); however, there are conflicting results as to whether lipid oxidation is enhanced or diminished in obese mice (Trajcevski et al., 2013). Our data recapitulate the effect of HFD on fasting blood glucose levels, such that the HFD had elevated basal circulating glucose compared to the LFD mice (Table 1), and this was confirmed by the glucose and insulin tolerances tests (Figure 4). These results suggest the HFD mice had significant insulin desensitization and impaired glucose uptake in peripheral tissues, namely skeletal muscle (DeFronzo & Tripathy, 2009; Thiebaud et al., 1982). These findings agree with previous studies, and strongly implicate impaired insulin action in skeletal muscle, as previous work suggests that at least 80 percent of glucose uptake occurs in skeletal muscle tissue (Thiebaud et al., 1982). In type 2 diabetic and obese mice, the loss of insulin sensitivity, or lowered insulin action, can impair metabolism and decrease mitochondrial function (Gupte et al., 2013). While succinic acid, or the ester form, has been suggested to simulate insulinotropic action (Fahien & MacDonald, 2002; MacDonald & Fahien, 1988), and thus glucose uptake and eventual aerobic metabolism, few studies have explored this phenomenon. Further, we observed that SA treatment increased fasting blood glucose, and the effect is more prominent in the HFD‐fed mice (Table 1). In contrast to our results, previous studies have found that succinate supplementation lowered basal fasting blood glucose levels and mitigated glucose intolerance (Mills et al., 2018). Moreover, previous studies have found that the genetic ablation of the succinate receptor (SUCNR1) defended against adipose tissue inflammation and glucose intolerance (van Diepen et al., 2017), and initial protection against obesity in HFD (McCreath et al., 2015) implicating succinic acid/SUCNR1 signaling in the development of obesity (Keiran et al., 2019; McCreath et al., 2015) and its consequences, though its effects can be positive (Keiran et al., 2019). Our data demonstrate that a high‐fat diet stimulates the expected hallmark obesity and an insulin‐resistant state but SA treatment had no clear positive effect. Thus, perhaps the ester forms of succinic acid may be beneficial in insulin‐mediated glucose disposal, but succinic acid, despite potential benefit on mitochondria, does not affect blood glucose regulation in diet‐induced obesity, or that only acute/intermittent treatments may prove beneficial.

### 4.4 The impact of SA treatment on mitochondrial respiratory capacity in diet‐induced obesity

Following diet‐induced obesity, skeletal muscle undergoes many adaptations including insulin resistance and altered lipid oxidation (He et al., 2001; Kelley et al., 1999; Kim et al., 2000). Due to the important role skeletal muscle plays in insulin‐stimulated glucose disposal, obesity and a HFD can negatively impact the mitochondrial content of the muscle and thus hinder its function (Hood, 2001; Wu & Ballantyne, 2017). Although previous work suggested that succinic acid treatment might ameliorate mitochondrial dysfunction (Ferro, Carbone, Marzouk, et al., 2017), our results from the glucose and insulin tolerance tests found that SA had no effect on the glucose tolerance or insulin sensitivity and thus did not rectify the HFD‐induced deficits (Figure 4). To assess the respiratory rates of each treatment group of mice and to explore the oxidative capacity and mitochondrial functioning, permeabilized whole soleus muscle tissue respiration was used. A previous study found that successfully permeabilized whole tissue preparations elicits high respiratory rates (Kuznetsov et al., 2008). Soleus muscle is also favorable to other muscles because it is highly oxidative, which allows specific intracellular energy transfer systems to remain in a tightly coupled state making the analysis of respiration less difficult (Kuznetsov et al., 2008). Further, the oxidative soleus muscle is suggested to be negatively impacted by high‐fat diet‐induced obesity, as obese mice have previously demonstrated an oxidative fiber type shift (Shortreed et al., 2009). Specifically, previous studies in mice have found that diet‐induced obesity increases type I and IIA muscle fiber cross‐sectional areas compared to normal chow (Shortreed et al., 2009; Trajcevski et al., 2013), demonstrating a significant muscle‐specific shift toward greater involvement of oxidative fibers and increased oxidative potential. Furthermore, shifts toward type I fiber content has been found to be an essential component of type 2 diabetes (Larsen et al., 2009). The shift to oxidative fibers due to a high‐fat diet‐induced obesity as well as the already highly oxidative state of the soleus tissue allows for it to be the optimal skeletal muscle to assess mitochondrial respiration and oxidative capacity. However, no work to date had explored the potential impact of succinic acid on the oxidative soleus muscle and its respiratory capacity.

In the present study, we observed no negative effect of a high‐fat diet on any respiratory state or specific mitochondrial complex in the soleus (Figure 5). Our findings contrast previous work which documented that high‐fat diet (McCreath et al., 2015; Özyazgan et al., 1998) and/or obesity (Gupte et al., 2013; Saravanan & Pari, 2006) can impair mitochondrial function. Though no significant differences were found between LFD and HFD, this observation indicates that both complexes I and II appear to be intact (Ferro, Carbone, Marzouk, et al., 2017). However, succinic acid treatment did increase specific activation of complex I driven respiration (Glu + Mal and ADP), complex II driven respiration (Succinate, ADP, and Rotenone, a complex I inhibitor), combined complex I + II driven respiration (Glu + Mal, ADP, and Succinate), and complex IV activity (A + TMPD, Figure 5). However, when antimycin A, a known complex III inhibitor, was assessed, there was a reduction in the complex III‐driven respiration compared to previous complex analysis, which was not different between diet or treatment. It is important to note that complex IV activity is known to correlate with citrate synthase, a classical indicator of mitochondrial content (Park et al., 2014), and electron microscopy‐based measure of mitochondrial content (Larsen et al., 2012). Thus, if the respiratory rates are normalized to complex IV activity, and thus mitochondrial content, the effect SA‐treatment is abolished, suggesting that the increased respiratory rates are the result of succinic acid‐induced mitochondrial biogenesis, and not a specific impact on the aforementioned mitochondrial complexes per se. Critically, respiratory rates were not increased with the addition of cytochrome C, which suggests maintained mitochondrial membrane integrity in all groups. These may be the first data to explore the impact of succinic acid treatment on skeletal muscle in HFD‐induced obesity and demonstrate the treatment may induce mitochondrial biogenesis. Though further work is needed to determine if this phenomenon is specific to muscle fiber type or tissue (e.g., adipose) and if more sophisticated measures of mitochondrial content (e.g., Electron Microscopy) agree.

### 4.5 Experimental considerations

The present study suggests that succinic acid treatment induced mitochondrial biogenesis with no diet‐induced dysfunction. The present study did not conduct any additional assessment of mitochondrial content, such as citrate synthase activity, though as mentioned previously complex IV activity correlates well with CS (Park et al., 2014) and electron microscopy‐based measures of mitochondrial content (Larsen et al., 2012), suggesting it is a representative indicator. Further, it has been suggested that CS activity can be acutely regulated by exercise and it may contribute to the oxidation of substrates in some respiratory procedures, thus may be problematic (Larsen et al., 2012). In the present study, given its relatively high oxidative capacity, the ease of whole tissue removal, and the glucose disposal that occurs in such skeletal muscle the respiratory rates of soleus muscle were assessed, though previous work has demonstrated that not all mitochondria function the same (Park et al., 2014), so future examination of the impact of succinic acid on different muscle fiber types is warranted. However, as previous research has found that diabetes affects skeletal muscle mitochondrial function and fiber expression only in the lower limbs, such as the soleus muscle (Larsen et al., 2009), we would argue this is a relevant model. Further, we used epididymal white adipose tissue mass as an indicator of adiposity, and more sophisticated measures of adiposity (e.g., MRS/MRI) were not available but should be employed in future investigations of the potential impact of succinic acid on HFD‐induced obesity. Further exploration of other adipose tissue depots, especially brown adipose tissue, is warranted to better understand the potential impacts of succinic acid treatment on adipose tissue mitochondrial capacity as recently described (Mills et al., 2018), and the potential influence on adipokines (e.g., IL‐6, TNFa, etc.). Finally, to reduce variability due to sex hormones, future studies should explore whether age or sex impacts the responses to succinic acid.

## 5 CONCLUSION

In a HFD‐induced model of obesity and insulin resistance, we found that treatment with the Krebs cycle intermediate succinic acid seems to decrease white adipose tissue mass, though did not impact caloric intake, body mass, physical activity, or glucose/insulin tolerance. In oxidative skeletal muscle of SA‐treated mice, there was increased respiratory capacity, owed to greater mitochondrial content, suggestive of succinate‐induced mitochondrial biogenesis. In conclusion, while succinic acid appears to induce favorable changes in mitochondria and adipose tissue mass, it does not positively affect blood glucose regulation.

## CONFLICT OF INTEREST

The authors have no conflicts of interest to report, financial or otherwise.

## AUTHOR CONTRIBUTIONS

SJI, SL, THR devised the study and methodology. SJI and THR supervised the experimental procedures and conducted statistical analyses. All authors performed the experimental procedures, data analysis, and revision of data analysis. SJI, KZ, and THR drafted the tables/figures and manuscript. All authors read, revised, and approved the manuscript.

## ETHICAL STATEMENT

As mentioned above, the local Institutional Animal Care and Use Committee at Skidmore College reviewed and approved the protocol, in accordance with Federal guidelines.

## Data Availability

Data available upon reasonable request from the corresponding author.
